# Comparative efficacy and safety between amisulpride and olanzapine in schizophrenia treatment and a cost analysis in China: a systematic review, meta-analysis, and cost-minimization analysis

**DOI:** 10.1186/s12888-018-1867-8

**Published:** 2018-09-05

**Authors:** Peng Men, Zhanmiao Yi, Chaoyun Li, Shuli Qu, Tengbin Xiong, Xin Yu, Suodi Zhai

**Affiliations:** 1 Department of Pharmacy, Peking University Third Hospital, N. Huayuan Rd, Beijing, China; 2 Institute for Drug Evaluation, Peking University Health Science Center, Beijing, China; 3 Department of Pharmacy Administration and Clinical Pharmacy, School of Pharmaceutical Science, Peking University, Beijing, China; 4 Health Economics & Outcome Research, Sanofi, Yanan Rd, Shanghai, China; 5 Real-World Insights, IQVIA, W. Beijing Rd, Shanghai, China; 6 Department of Psychiatry, Peking University Sixth Hospital, N. Huayuan Rd, Beijing, China

**Keywords:** Amisulpride, Olanzapine, Schizophrenia, Meta-analysis, Cost-minimization analysis, China

## Abstract

**Background:**

Amisulpride was introduced into China in 2010 as a second-generation atypical antipsychotic, while olanzapine has been on the market since 1999 as one of the leading treatments for schizophrenia in China. Since more Chinese patients are gaining access to amisulpride, the study aims to compare the efficacy, safety, and costs between amisulpride and olanzapine for schizophrenia treatment in China.

**Methods:**

PubMed, Embase, the Cochrane library, China National Knowledge Infrastructure (CNKI) and WanFang database were systematically searched for randomized controlled trials (RCTs) up to July 2018. The Cochrane Risk of Bias tool was utilized to assess the quality of included studies. A meta-analysis was performed to compare the efficacy and safety of amisulpride and olanzapine, followed by a cost-minimization analysis using local drug and medical costs reported in China.

**Results:**

Twenty RCTs with 2000 patients were included in the systematic review. There were no significant differences between amisulpride and olanzapine on efficacy measures based on scores from the Positive and Negative Syndrome Scale, the Scale for the Assessment of Negative Symptoms, the Brief Psychiatric Rating Scale and the Clinical Global Impressions-Severity or Improvement. For safety outcomes, amisulpride was associated with lower fasting blood glucose and less abnormal liver functions as well as significantly lower risks of weight gain, constipation, and somnolence; olanzapine was associated with significantly lower risks of insomnia and lactation/amenorrhea/sexual hormone disorder. No significant differences were found in risks of extrapyramidal symptoms (EPS), tremor, akathisia, abnormal corrected QT interval. Cost-minimization analysis showed that amisulpride was likely to be a cost-saving alternative in China, with potential savings of 1358 Chinese Yuan (CNY) per patient for a three-month schizophrenia treatment compared with olanzapine.

**Conclusion:**

As the first meta-analysis and cost-minimization analysis comparing the efficacy, safety and cost of amisulpride and olanzapine within a Chinese setting, the study suggests that amisulpride may be an effective, well-tolerated, and cost-saving antipsychotic drug alternative in China.

## Background

As a chronic and severe mental disorder, schizophrenia is associated with psychotic behaviors such as hallucinations, delusions, thought/movement disorders which are not generally seen in healthy people (positive symptoms), and disruptions to normal emotions and behaviors (negative symptoms) [1]. Schizophrenia patients may also suffer from functional impairments that consequently affect their social relationships and employment opportunities.

Globally, schizophrenia affected more than 23 million people in 2015. It was among the top ten causes of disability in adolescents and young adults aged between 15 and 39 [2]. A previous study estimated its prevalence in China and reported a two-fold increase in the number of patients, which increased from 3.09 million in 1990 to 7.16 million in 2010 [3]. Another meta-analysis [4] estimated that the lifetime prevalence of schizophrenia among Chinese people was 5.44 per 1000 in 2014, which means that there were 7.4 million patients with schizophrenia in China (the Chinese population was 1.364 billion in 2014). Also, the stigma faced by schizophrenia patients as well as their families is still common in Chinese society, therefore often impeding the access and continuity of disease treatment [5].

The economic burden of schizophrenia is heavy in China. The chronically debilitating course of disease and relapses during early and late stages of schizophrenia imply the requirement for long-term continuous treatment, which imposes a considerable economic burden on healthcare systems [6]. Total direct and indirect costs for schizophrenia treatment in China range from 94 million to 102 billion US dollar annually [7]. Data obtained from the Tianjin Urban Employee Basic Medical Insurance (UEBMI) showed an annual, schizophrenia-related, mean direct cost of 1775 US dollar per patient, with hospitalized patients spending much more than non-hospitalized patients [8].

Antipsychotics are the core treatment measures for schizophrenia [9]. Atypical antipsychotics, also known as second-generation antipsychotics, are the most commonly prescribed medications. Among them, amisulpride was introduced into China in 2010, while olanzapine has been on the market since 1999 and is currently one of the leading treatments for schizophrenia in China. Olanzapine developed quickly after entering the Chinese market, with annual sales rising from less than 40 million Chinese Yuan (CNY) in 2005 to 420 million CNY in 2014; during this period, the compound annual growth rate for olanzapine reached 30% [10].

Studies have been conducted to investigate amisulpride in comparison with other atypical antipsychotics in different populations. A Cochrane systematic review published in 2010 summarized corresponding clinical outcomes and found that the efficacy of amisulpride was similar to that of olanzapine and risperidone, and better than that of ziprasidone. Amisulpride was likely to be associated with less weight gain than risperidone and olanzapine [11]. However, the review contained no studies performed on Chinese patients. Ever since amisulpride was introduced into China in 2010, numerous studies comparing various efficacy and safety outcomes between amisulpride and olanzapine have been performed. Since an increasing number of Chinese patients have potential access to amisulpride, it is necessary to synthesize the available evidence through a systematic review and meta-analysis.

Given the economic burden of schizophrenia, the medical costs of amisulpride and olanzapine treatment are also of vital importance. Moreover, local economic evidence is increasingly required and used by relevant authorities in China during pricing and reimbursement decision-making. Appropriate treatment choices may reduce patient financial burden as well as medical insurance costs. Although economic analyses of atypical antipsychotics have been performed in some countries, China has yet to do the same [12–14].

Therefore, the objectives of this study were to 1) summarize and investigate the clinical efficacy and safety profiles of amisulpride and olanzapine as schizophrenia treatments through a systematic and repeatable evaluation of both Chinese and foreign randomized-controlled trials (RCTs); and 2), assess the costs of amisulpride and olanzapine (from a healthcare system perspective) for the treatment of schizophrenia in China.

## Methods

### Systematic review

The methods for systematic review adhered to guidelines published by Cochrane Collaboration [15] and the UK’s National Institute for Health and Clinical Excellence (NICE) [16]. NICE has a rigorous specified approach to perform systematic reviews, and the standard is generally considered sufficient by other countries’ health technology assessment agencies. The systematic review with meta-analysis was registered on PROSPERO (No. CRD 42017069524).

To identify the relevant published studies for clinical data, PubMed, Embase and the Cochrane Library were searched for RCT literature in English, and the China National Knowledge Infrastructure (CNKI) and WanFang database were searched for RCTs references in Chinese. RCTs with head-to-head comparisons between amisulpride and olanzapine were of interest. In English databases, we combined disease terms (schizophrenia) AND intervention terms (amisulpride AND olanzapine) AND study design terms (RCT, clinical trials) AND outcomes. The search terms were translated into Chinese when we searched CNKI and WanFang database. Searches were conducted separately for each literature database up to January 2017. A complementary search was performed in order to cover the most recent articles (published before July 2018). Reference lists of identified studies and relevant systematic reviews were also screened to ensure that relevant items were not missed in the search. The scope of each search strategy has been defined and reported in the PICOS statements in Table 1.

**Table 1:** PICOS statement for inclusion and exclusion criteria

	Inclusion Criteria	Exclusion Criteria
Study population	Schizophrenia patients, regardless of their age, sex, ethnic group or disease status.	Any not listed in the inclusion criteria
Intervention	Amisulpride in any oral form of application with any dose	Any not listed in the inclusion criteria
Comparator	Olanzapine in any oral form of application with any dose	Any not listed in the inclusion criteria
Outcome measures	Clinical efficacy outcomes Safety outcome	Any not listed in the inclusion criteria
Study design	RCTs with head-to-head comparison	Editorials OR Notes OR Comments OR Letters OR Case reports OR Pharmacokinetic studies OR Epidemiology studies
Restrictions	Full-text published manuscripts in English or Chinese Year limitation: no limit	Duplicates Not full-text published manuscripts Non-English or non-Chinese studies

Two reviewers (MP, YZ) independently extracted all the data. The disagreement was solved by discussion and further inspection of articles. A data extraction table was developed in Microsoft Excel to integrate data from included trials. General information regarding the identification of publication, such as author, title, year of publication, and study design were extracted. In addition, data on sample size (completed/all enrolled), patient characteristics, treatment arm characteristics, outcomes of efficacy and safety were also documented. For studies with multiple arms, only information on amisulpride and olanzapine groups was extracted.

Clinical efficacy endpoints were assessed by the mean change from baseline to total score for four common rating scales of schizophrenia: Positive and Negative Syndrome Scale (PANSS), Scale for the Assessment of Negative Symptoms (SANS), the Brief Psychiatric Rating Scale (BPRS), and the Clinical Global Impressions - Severity or Improvement (CGI-SI). Regarding PANSS, the proportion of patients were also defined as “improved” (25% to no more than 50% reduction in PANSS score), “much improved” (50% to no more than 75% reduction in PANSS score), or “very much improved” (more than 75% reduction in PANSS score).

Safety outcome endpoints included extrapyramidal symptoms (EPS), weight gain, metabolic parameters (total cholesterol, high-density lipoprotein (HDL), low-density lipoprotein (LDL), triglycerides and blood glucose), headache, insomnia, somnolence, xerostomia, increased salivation, constipation, hypotension and abnormal corrected QT interval (QTc).

The Cochrane Collaboration’s tool for assessing risk of bias was employed to assess the methodological quality of included RCTs [17]. Two reviewers independently assessed six domains, which comprised of selection bias (random sequence generation and allocation concealment), performance bias (blinding of participants and personnel), detection bias (blinding of outcome assessment), attrition bias (incomplete outcome data), reporting bias (selective reporting) and other sources of biases. The risk of bias in each domain was categorized as “low”, “high” or “unclear”.

### Meta-analysis

Risk ratios (RRs) and weighted mean differences (WMDs) with 95% confidence intervals (CIs) were calculated for dichotomous and continuous outcomes respectively. A *p* < 0.05 was considered statistically significant. Meta-analysis was performed using STATA (version 12; Stata Corp., College Station, Texas, USA). Heterogeneity was assessed by Cochran’s Q statistic and *I*^*2*^ statistic. The significance of the Q-statistic test with *P* < 0.10 indicated a substantial level of heterogeneity. The *I*^*2*^ statistic revealed that the percentage of variability in effect estimates was the result of heterogeneity rather than sampling error, where *I*^*2*^ values of 50% or more implied a substantial level of heterogeneity. The fixed effects model was utilized for *I*^*2*^ values lower than 50% and the Q-statistic test *p* values greater than 0.10; otherwise, the random effects model was used. The pooled results were displayed using forest plots.

### Cost-minimization analysis

Following the meta-analysis, a Microsoft Excel-based cost-minimization analysis (CMA) was conducted to compare the local drug and medical costs between amisulpride and olanzapine for schizophrenia patients in China.

IQVIA China Hospital Pharmaceutical Audit (CHPA) database served as the primary data source for retrieving associated drug acquisition costs in China’s setting. The CHPA database reports the market purchase prices at which the panel hospitals purchase products from wholesalers, distributors, and manufacturers. Database findings showed that the unit costs of original and generic drugs varied greatly in China. Because both Chinese and foreign studies were included in the meta-analysis, and several Chinese studies used generic amisulpride and olanzapine in the trials, we conducted the CMA in two scenarios in order to differentiate the impact of original and generic drug costs. In the base case analysis, we applied weighted-average drug costs for both original and generic drugs, while in an additional scenario, we only applied the average cost of original drugs in the model. All resource costs were represented in CNY.

Weighted-average dosages from included trials of the systematic review were calculated and applied in the CMA. The time horizon of the CMA was based on the average duration of follow-up of included studies.

As for medical costs, the assumption was made that the efficacy and hospitalization rates between both treatments were equivalent; only costs of the adverse events were brought into the analysis. Costs of adverse events were collected from a panel of local clinical experts. As suggested by the clinical experts, in real-world practice, the treatment rates for some of the adverse events (e.g. somnolence, constipation, insomnia) are very low, thus we only considered the cost to treat adverse events that had clinical significance. Probabilities of adverse events were calculated based on the number of events and totals from included trials.

In both the base case scenario and the additional scenario, sensitivity analyses were performed accordingly to examine the stability and robustness of the results. In the univariate sensitivity analysis, unit costs and probabilities of adverse events varied by ±20%. Since daily dosages of antipsychotics vary considerably between different stages of disease progression, we used a large range – 400-800 mg for amisulpride and 5-20 mg for olanzapine – as recommended by Chinese treatment guidelines [9]. In the probabilistic sensitivity analysis, Monte Carlo simulations (1000 times) were performed to test the uncertainty in the base case scenario. Cost differences were calculated and presented in frequency distributions and the probability of which amisulpride was still cost-saving.

## Results

**Fig. 1:**
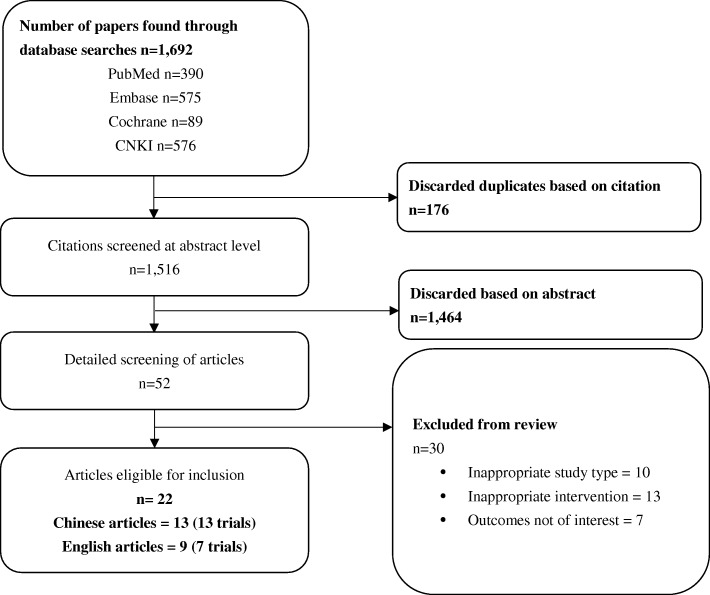
Selection process for articles in the systematic review

**Fig. 2:**
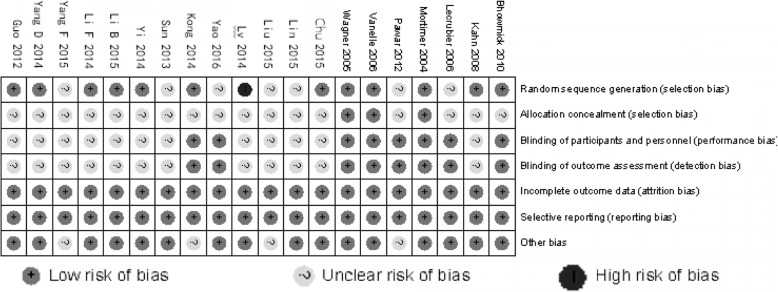
Risk of bias assessment for included studies

**Fig. 3:**
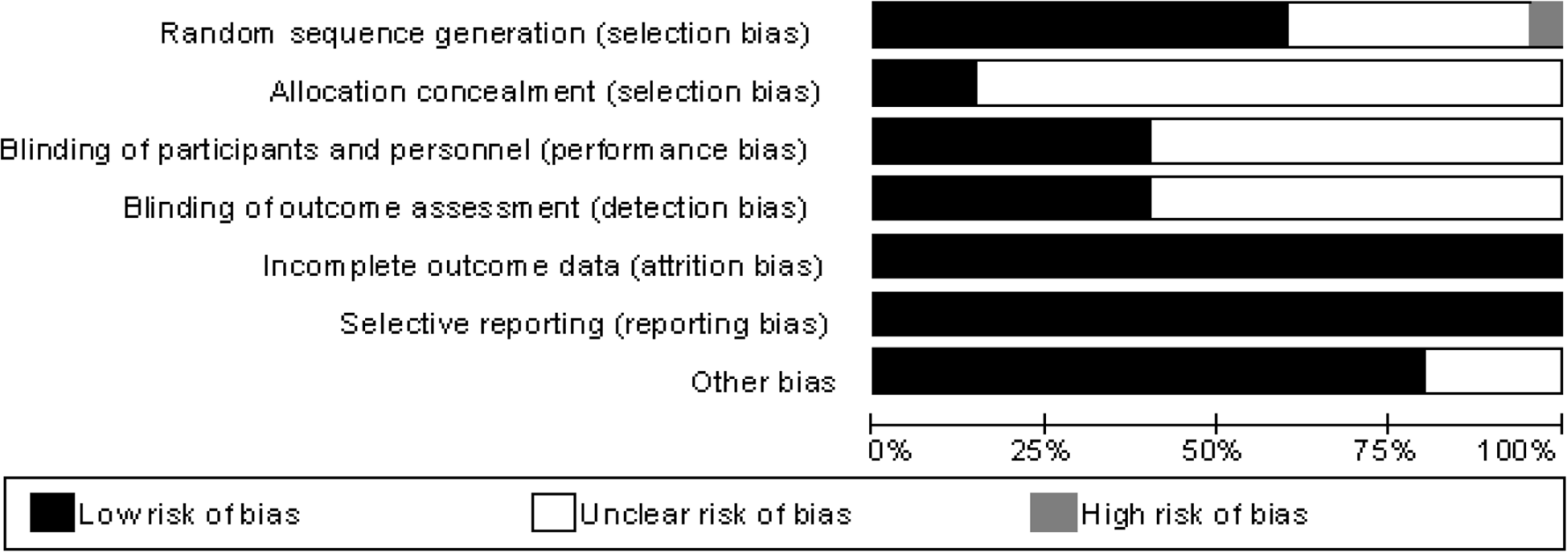
Summary of risk of bias assessment

Twenty RCTs with 2000 patients involving treatment with either amisulpride or olanzapine were identified and included as a result of the systematic review. Among these 20 trials, 13 (65.0%) were Chinese studies. The process of study selection and the final results of the search are illustrated below using the PRISMA Flow Diagram in Fig. 1. The treatment groups in each included trial were generally balanced with respect to demographic and clinical characteristics (Table 2). Each enrolled participant had an explicit diagnosis of schizophrenia based on the definitions in the Diagnostic and Statistical Manual of Mental Disorders, 4th Edition (DSM-IV), International Statistical Classification of Diseases and Related Health Problems 10th Revision (ICD-10), Chinese Classification and Diagnosis of Mental Diseases-3rd edition (CCMD3), or Mini International Neuropsychiatric Interview Plus (MINI-plus) criteria. The results and summary of risk of bias assessment for included studies were summarized in Figs. 2 and 3. The majority of included studies possessed low and/or moderate risk of bias. Selection bias (e.g. unclear random sequence generation, allocation concealment or blinding) was the dominant cause of high and unclear risk of bias.

**Table 2:** Baseline characteristics of included trials

Study	Country	Dosage **r**ange (mg/d)		Sample size (Completed/All enrolled)		F/U (weeks)	Mean age(y)	Gender (M, %)	Diagnosis criteria	Randomization	Blinded	Disease state	Comorbidity
		Amisulpride	Olanzapine	Amisulpride	Olanzapine								
Bhowmick 2010 [20]	India	100–800	10–20	39/40	38/40	12	31.6	53.2%	DSM-IV	Y(computer-generated random number list)	Single-blind	/	/
Chu 2015 [21]	China	300–600	10–20	19/20	18/20	12	26.9	56.4%	ICD-10	Y(random number table)	N	First episode	/
Guo 2012 [22]	China	600–1200	10–20	38/40	39/40	8	30.0	58.8%	CCMD-3	Y(random number table)	N	/	/
Kahn 2008 [23]	Multiple countries	200–800	5–20	69/104	82/105	52	25.8	59.8%	MINI plus	Y(a dedicated web-based online system)	N	/	/
Kong 2014 [24]	China	400–1000	15–20	35/35	35/35	8	29.5	61.4%	CCMD-3	Y(random digital coding)	N	First episode	/
Lecrubier 2006 [18]	France	150	20	67/70	64/70	24	37.0	72.9%	DSM-IV	Y	double-blind	chronic schizophrenia (≥1 yr)	/
Li B 2015 [25]	China	800	20	40/40	40/40	12	41.1	60.0%	CCMD-3	Y(random number table)	N	First episode	/
Li F 2014 [26]	China	200–800	10–20	31/31	31/31	8	27.7	54.8%	DSM-IV	Y(random number table)	N	First episode	/
Lin 2015 [27]	China	600–800	10–20	30/32	31/32	8	31.3	100.0%	ICD-10	Y	N	acute schizophrenia	/
Liu 2015 [28]	China	400–800	10–20	22/25	23/25	6	34.3	0.0%	ICD-10	Y	N	/	/
Lv 2014 [29]	China	200–800	5–20	40/40	40/40	8	35.9	0.0%	CCMD-3	Y(admission order)	N	/	/
Mortimer 2004 [30]	Multiple countries	200–800	5–20	117/189	125/188	24	37.8	65.0%	DSM-IV	Y(computer-generated random number list)	double-blind	acute schizophrenia	/
Pawar 2012 [31]	India	400–800	10–20	32/40	32/41	8	30.3	54.7%	ICD-10	Y	double-blind	First episode	/
Sun 2013 [32]	China	800–1200	15–20	36/38	32/36	6	27.3	51.4%	DSM-IV	Y	N	acute schizophrenia	/
Vanelle 2006 [33]	Multiple countries	200–600	5–15	35/45	36/40	8	34.4	63.5%	DSM-IV	Y(computer-generated random number list)	double-blind	/	Depression
Wagner 2005 [34]	Germany	400–800	10–20	18/26	18/26	8	36.3	36.1%	DSM-IV, ICD-10	Y(pseudo-random computer algorithm)	double-blind	/	/
Yang D 2014 [35]	China	200–1200	5–20	61/63	60/63	12	33.2	50.4%	CCMD-3	Y(random number table)	N	/	/
Yang F 2015 [36]	China	100–300	10–20	42/42	43/43	12	36.1	49.4%	ICD-10	Y	N	/	/
Yao 2016 [37]	China	800–1200	15–20	30/30	35/35	8	28.3	47.7%	ICD-10	Y	Single-blind	First episode	/
Yi 2014 [38]	China	max.1000	max. 20	29/30	30/30	8	70.4	55.0%	DSM-IV	Y(random number table)	N	/	/

Note: Included studies that published in English did not mention whether the amisulpride medication was generic or original, while five Chinese studies reported the information, all of which were generic

*F/U* Follow up, *DSM-IV* Diagnostic and statistical manual of mental disorders, 4th edition, *ICD-10* International statistical classification of diseases and related health problems 10th revision, *CCMD-3* Chinese classification and diagnosis of mental diseases-3rd edition, *MINI plus* Mini international neuropsychiatric interview plus

### Comparison of amisulpride and olanzapine on efficacy

**Fig. 4:**
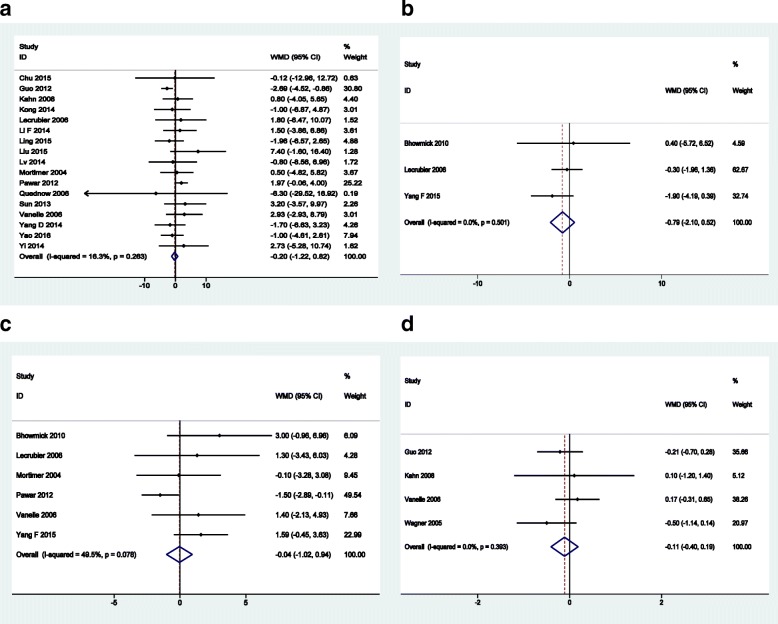
Forest plots of total scores for four common rating scales of schizophrenia: **a** PANSS; **b** SANS; **c** BPRS; **d** CGI-SI

The efficacy profile of olanzapine was similar to that of amisulpride. The results of mean changes from baseline in the PANSS total score showed no difference between amisulpride and olanzapine groups (20 trials, WMD = − 0.20, 95%CI: -1.22 to 0.82, Fig. 4).

**Fig. 5:**
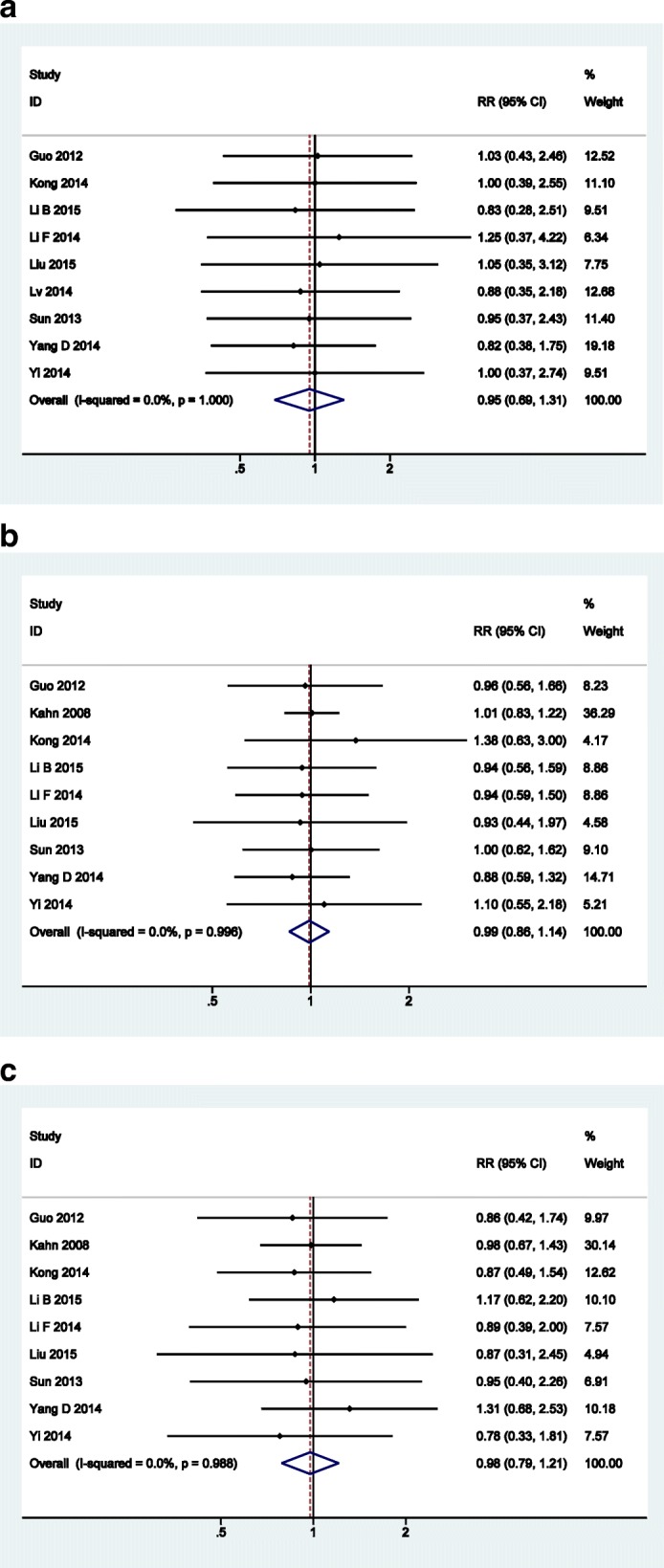
Forest plots of proportion of patients defined as **a** improved, **b** much improved and **c** very much improved (based on PANSS)

The proportions of patients assessed as “improved” (RR = 0.95, 95%CI: 0.69 to 1.31), “much improved” (RR = 0.99, 95%CI: 0.86 to 1.14), and “very much improved” (RR = 0.98, 95%CI: 0.79 to 1.21) were also similar between amisulpride and olanzapine groups (Fig. 5).

Meanwhile, there were no significant difference in effect sizes between the two treatment groups in terms of mean changes from baseline in SANS (WMD = − 0.79, 95%CI: -2.10 to 0.52), BPRS (WMD = − 0.04, 95%CI: -1.02 to 0.94), and CGI-SI (WMD = − 0.11, 95%CI: -0.40 to 0.19) total scores. The forest plots of efficacy results are presented in Fig. 4.

### Comparison of amisulpride and olanzapine on safety

**Fig. 6:**
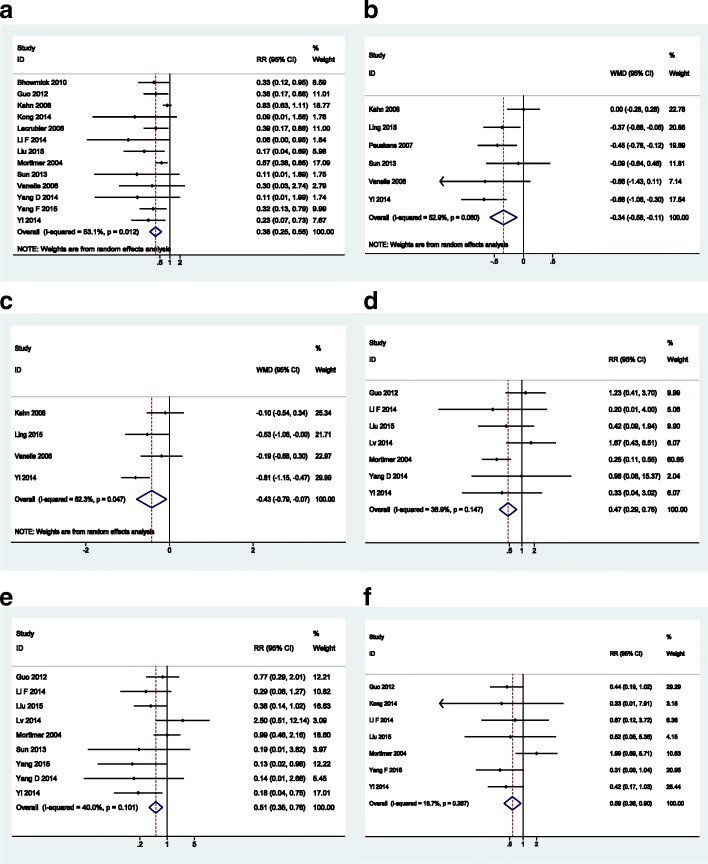
Forest plots for of safety outcomes that favor amisulpride: **a** weight gain; **b** blood glucose; **c** total cholesterol; **d** abnormal liver function; **e** somnolence; **f** constipation

When comparing amisulpride with olanzapine, the differences were statistically significant among the two patient groups. Amisulpride-treated patients experienced more weight gain over their baseline body weight (RR = 0.38, 95%CI: 0.25 to 0.56), decreased blood glucose (WMD = − 0.34 mmol/L, 95%CI: -0.58 to − 0.11) and lowered total cholesterol (WMD = − 0.43 mmol/L, 95%CI: -0.79 to − 0.07). Amisulpride was also significantly superior to olanzapine with lower risks of abnormal liver function (liver transaminase elevation, RR = 0.47, 95%CI: 0.29 to 0.75), somnolence (RR = 0.51, 95%CI: 0.35 to 0.76) and constipation (RR = 0.59, 95%CI: 0.38 to 0.90). The forest plots of safety outcomes that favor amisulpride are presented in Fig. 6.

**Fig. 7:**
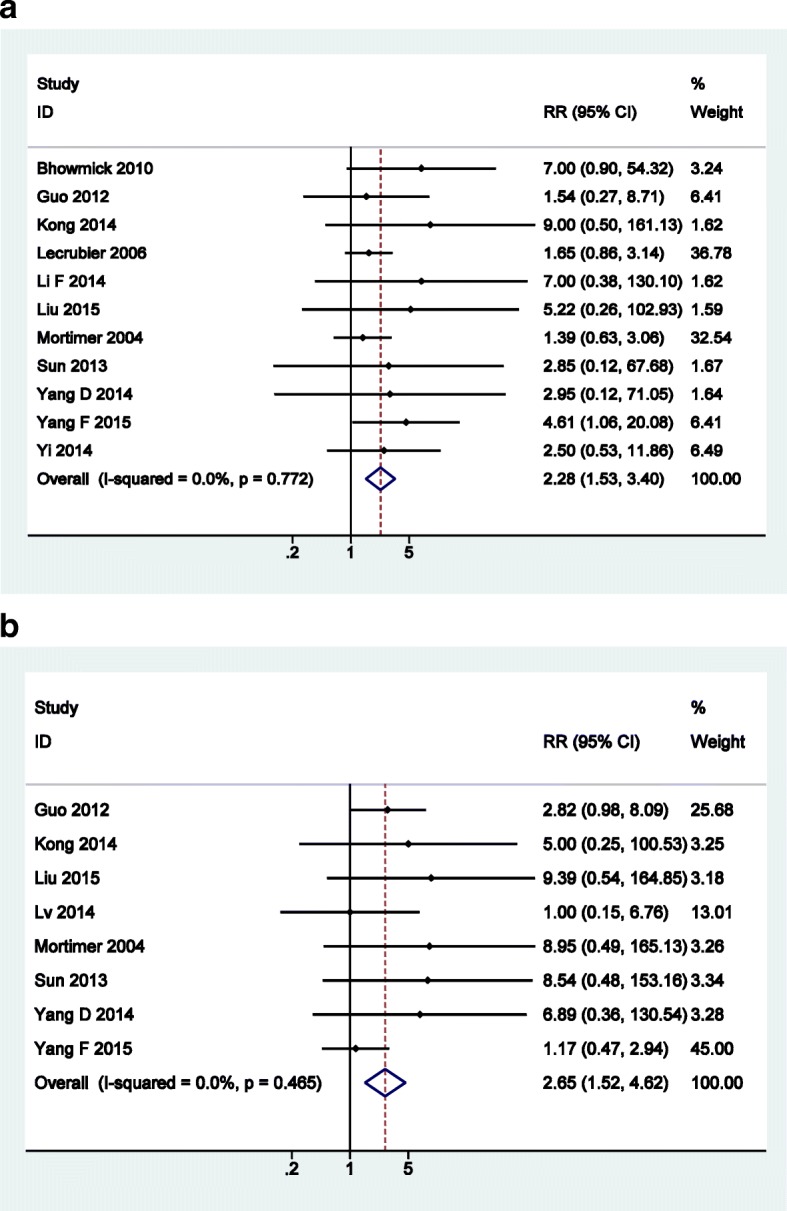
Forest plots for of safety outcomes that favor olanzapine: **a** insomnia; **b** lactation/amenorrhea/sexual hormone disorder

On the other hand, amisulpride induced significant higher risks of insomnia (RR = 2.28, 95% CI: 1.53 to 3.40) and lactation/amenorrhea/sexual hormone disorder (RR = 2.65, 95%CI: 1.52 to 4.62). The forest plots of safety outcomes that favor olanzapine are presented in Fig. 7.

**Fig. 8:**
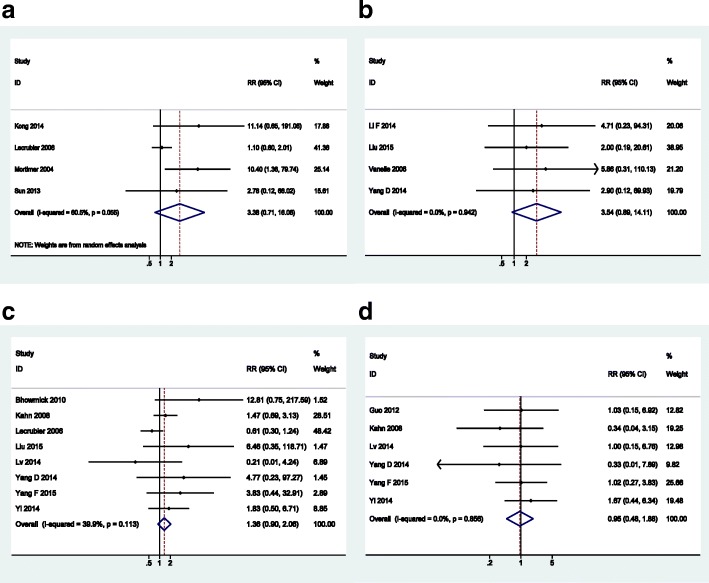
Forest plots of outcomes without statistical significance: **a** EPS; **b** tremor; **c** akathisia; **d** abnormal QTc

For patients treated with amisulpride, the incidences of EPS (RR = 3.38, 95%CI: 0.71 to 16.06), akathisia (RR = 1.36, 95%CI: 0.90 to 2.06) and tremor (RR = 3.54, 95%CI: 0.89 to 14.11) were higher than those of patients with olanzapine but with no statistical significance. Other AEs such as HDL, LDL, triglycerides, headache, xerostomia, increased salivation, hypotension and abnormal QTc were similar between the two groups. The forest plots of outcomes without statistical significance are presented in Fig. 8.

### Cost-minimization analysis comparing amisulpride and olanzapine

Among 13 included trials in China, 7 reported average doses of amisulpride and olanzapine during the treatment periods. Weighted average dosages from the 7 trials were 551.11 mg per day for amisulpride and 12.73 mg per day for olanzapine (Table 3). Average follow-up time in the 13 included Chinese studies was 3 months. The probabilities of adverse events, costs of adverse events and ranges tested for univariate sensitivity analysis are displayed in Table 4.

**Table 3:** Included Chinese studies that reported average daily dosage

Study	Amisulpride		Olanzapine	
	n	Avg. dosage(mg/d)	n	Avg. dosage(mg/d)
Yang 2015 [36]	42	222.36	43	15.27
Yi 2014 [38]	29	503.45	29	8.50
Lv 2014 [27]	40	642.50	40	14.12
Yang 2014 [33]	61	600.00	60	10.00
Guo 2012 [20]	38	857.20	39	15.00
Chu 2015 [21]	19	465	18	16.6
Lin 2015 [27]	30	503	31	11.57
Weight average daily dosage	551.11		12.73

**Table 4:** Cost-minimization analysis comparing amisulpride and olanzapine in the treatment of Chinese patients with schizophrenia

Data Input	Amisulpride		Olanzapine	
Drug costs	Mean	Range	Mean	Range
Unit drug cost – base case (CNY)	8.40	6.72–10.08	15.05	12.04–18.06
Unit drug cost – scenario (CNY)	13.53	10.82–16.23	21.90	17.52–26.28
Daily dosage (mg)	565.78	400.00–800.00	12.57	5.00–20.00
Probability of adverse events				
Probability of weight gain	0.15	0.12–0.18	0.40	0.32–0.48
Probability of increased blood glucose levels	0.03	0.02–0.03	0.06	0.04–0.07
Probability of liver function damage	0.06	0.04–0.07	0.13	0.1–0.16
Probability of lactation/amenorrhea/sexual hormone disorder	0.09	0.07–0.11	0.03	0.02–0.03
Cost of adverse events (CNY)	Mean	Range	Data Source	
Weight gain	29	23.2–34.8	KOL interview^a^	
Increased blood glucose levels	2111	1688.8-2533.2	KOL interview	
Liver function damage	575	460–690	KOL interview	
Lactation/amenorrhea/sexual hormone disorder	352	281.6–422.4	KOL interview	

^a^*KOL* Key opinion leader

#### Base case scenario

In the base case scenario, we used the weighted average cost of both original and generic drugs. Unit costs per 200 mg amisulpride and 5 mg olanzapine were 8.40 CNY and 15.05 CNY, respectively. The estimated 3-month costs per patient were 2273 CNY for amisulpride and 3615 CNY for olanzapine, with an incremental saving of 1358 CNY per patient.

#### Additional scenario

In the additional scenario using original drug costs, unit costs per 200 mg amisulpride and 5 mg olanzapine were 13.53 CNY and 21.90 CNY, respectively. The estimated 3-month costs per patient were 3567 CNY for amisulpride and 5168 CNY for olanzapine, with an incremental cost of 1601 CNY per patient.

#### Sensitivity analyses

**Fig. 9:**
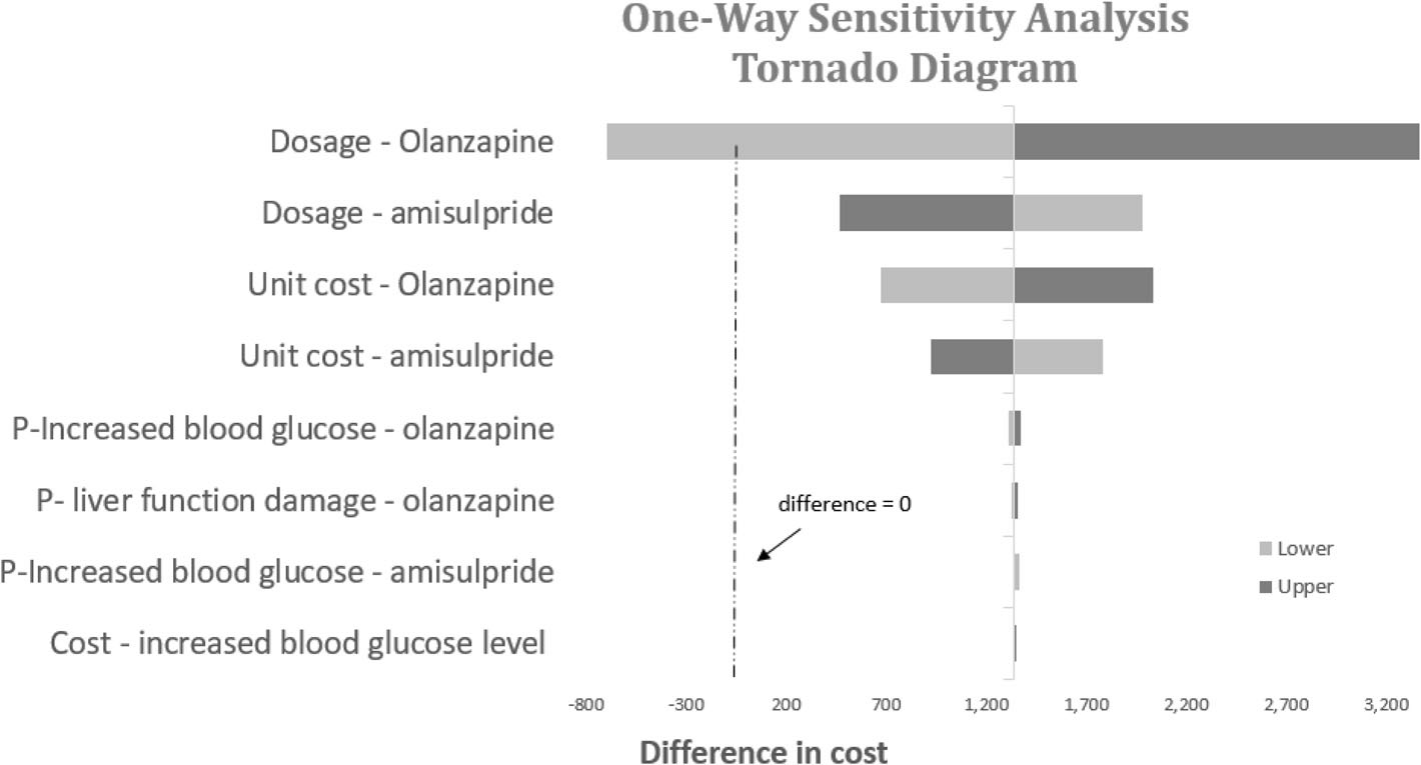
Tornado diagram for one-way sensitivity analysis of cost-minimization analysis (base case)

**Fig. 10:**
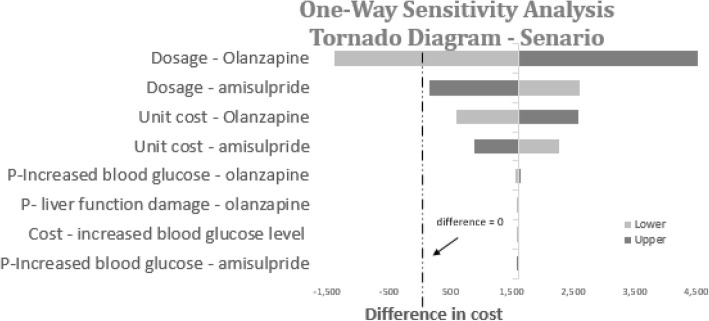
Tornado diagram for one-way sensitivity analysis of cost-minimization analysis (scenario)

Results of the univariate sensitivity analyses are displayed in tornado diagrams for both the base case scenario (Fig. 9) and the additional scenario (Fig. 10). Based on our findings, dosage and unit costs of amisulpride and olanzapine demonstrated the largest impacts on cost differences. Amisulpride was consistently seen as the more cost-saving alternative except when the comparison group used the lowest daily dosage (5 mg) of olanzapine. Although Chinese treatment guidelines [9] recommend a large dosage range, it is noteworthy to acknowledge that a 5 mg -dosage for olanzapine is rare in a real-world setting. In the included Chinese studies, the lowest olanzapine dosage was 8.5 mg daily.

**Fig. 11:**
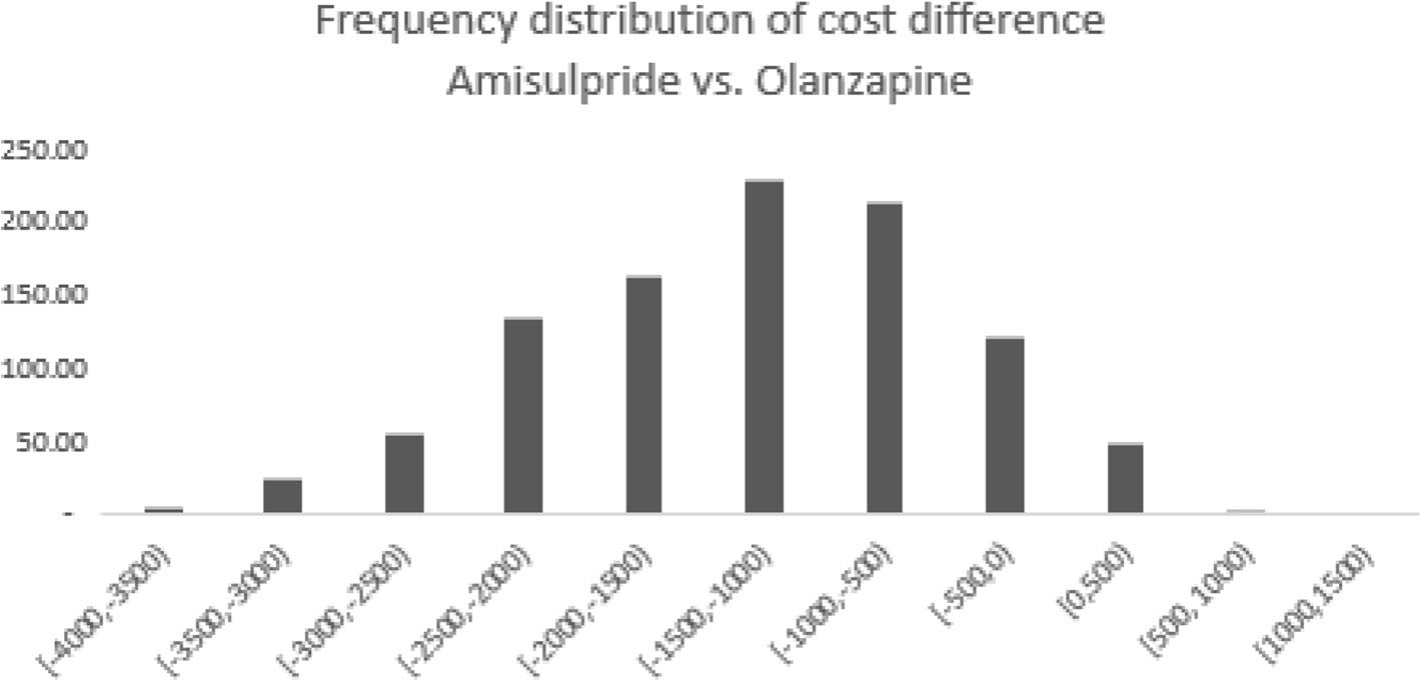
Frequency distribution of 3-month cost difference for the probabilistic sensitivity analysis

The probabilistic sensitivity analyses demonstrated a 94.6% probability that amisulpride is a cost-saving alternative, thus confirming the stability and robustness of the base case analysis. Frequency distributions of cost differences are presented in Fig. 11.

## Discussion

In this comprehensive systematic review and meta-analysis of head-to-head clinical trials between amisulpride and olanzapine, both drugs were similar in terms of treatment efficacy for schizophrenia. There were no significant differences in mean changes from baseline for total scores of PANSS, SANS, BPRS, and CGI-SI between patients treated with these two drugs. The proportion of patients assessed as improved, much improved, and very much improved were also similar between the groups. Additionally, one included trial [18] showed no statistical difference between amisulpride and olanzapine groups on relapse rates. These results were consistent with those in previous systematic reviews and meta-analyses [11]. Other efficacy outcomes such as quality of life (QoL) and general function are also important. However, only six studies reported related outcomes and used different measurement tools respectively. Thus, we did not include the QoL and general function outcomes in our analyses.

In terms of safety and tolerability, amisulpride and olanzapine were both tolerable and showed different characteristics on specific outcomes. Our analyses revealed that amisulpride had significantly more favorable effects than olanzapine for weight gain, blood glucose, and total cholesterol, which indicated a better influence on metabolic parameters. A previous study found a dose-dependent weight gain after 6 weeks of olanzapine treatment due to its antagonism at 5-HT_2C_ and H1 receptors. The results of our study implied that patients with a family or personal history of diabetes, dyslipidemia and obesity should be cautious and attentive to their body weights, fasting blood glucose levels and lipid profiles before starting and receiving olanzapine treatment [19].

Amisulpride also demonstrated superiority for lower risks of constipation, liver transaminase elevation levels and somnolence, while its risks on insomnia and lactation/ amenorrhea/sexual hormone disorder were significantly higher than that of olanzapine. The results were consistent with a previous systematic review [11]. Concerning risks of EPS, akathisia and tremor, although olanzapine showed better safety profile, the results were not significant. Olanzapine showed a better safety profile for EPS, akathisia and tremor, however the results were not significant. More large-scale trials are required to further investigate the differences between the adverse events for amisulpride and olanzapine.

Our study compared amisulpride and olanzapine for the treatment of schizophrenia by analyzing a large number of RCTs. Although one trial included a mix of patients with schizophrenia, schizophreniform and schizoaffective disorder (of which 57% were schizophrenia patients), this particular trial was nevertheless selected for our study due to its good quality and large sample size. To justify the trial’s inclusion, we conducted sensitivity analyses to test the impact of this trial against others, results of which showed no significant differences among all of the outcomes.

Cost minimization analysis showed that amisulpride might act as a cost-saving alternative to olanzapine in the local Chinese setting with a potential saving of 1358 CNY every 3 months for a patient with schizophrenia in the acute phase, and further potential savings in the maintenance phase. In the additional scenario when original drug costs were applied, the potential savings increased to 1601 CNY per patient. Sensitivity analyses demonstrated the robustness of the results, with dosage and unit costs of amisulpride and olanzapine identified as the most sensitive factors.

The funding sources of included studies were also verified. Three of the 13 Chinese studies reported their funding sources as hospitals. Among the 7 foreign studies, 5 reported their sponsorships: Two studies were from Eli Lilly, one study from Sanofi, one from Sun Pharma, and one from a joint grant from AstraZeneca, Pfizer and Sanofi. All eight studies declared that the sponsor(s) exerted no influence on the results of the studies.

This study is the first meta-analysis of amisulpride to include data from Chinese patients and may therefore be used to inform better treatment decision-making as China gains increasing access to amisulpride and olanzapine. Moreover, it is the first study to compare the economic benefits of both medications.

Despite the usefulness of this study, limitations remain. First, as there are several different outcome scales used for schizophrenia patients, data from some scales in the included studies were limited (for example, only one study provided data of relapse rate), thus making it difficult to carry a better meta-analysis with these scales. Secondly, although a majority of included studies carried low and moderate risk of bias, the quality of included Chinese studies was lower than that of foreign studies, especially in terms of unclear bias in random sequence generation, allocation concealment and blinding. High-quality research on Chinese patients is required to assess clinical practices and support guideline recommendations. Thirdly, the heterogeneity of population (such as sex and age differences) included in the trials may affect the efficacy and safety results. Finally, since published cost data for the treatment of adverse events is currently unavailable, cost data were retrieved from clinical expert opinion, which oftentimes require further confirmation by chart reviews, clinical trials or real-world studies. Only drug costs and costs for selected adverse events were taken into account; social costs such as sick absence and unemployment were not considered in the CMA.

## Conclusion

This study suggests that amisulpride is an effective and well-tolerated antipsychotic drug, and may act as a cost-saving alternative to olanzapine in China. The results may provide an important reference for clinical decision-making in China.

## Abbreviations

BPRS: Brief Psychiatric Rating Scale
CCMD3: Chinese Classification and Diagnosis of Mental Diseases-3rd edition
CGI-SI: Clinical Global Impressions - Severity or Improvement
CHPA: IQVIA China Hospital Pharmaceutical Audit
CI: Confidence interval
CMA: Cost-minimization analysis
DSM-IV: Diagnostic and Statistical Manual of Mental Disorders, 4th Edition
EPS: Extrapyramidal symptoms
HDL: High-density lipoprotein
ICD-10: International Statistical Classification of Diseases and Related Health Problems 10th Revision
LDL: Low-density lipoprotein
MINI-plus: Mini International Neuropsychiatric Interview Plus
NICE: UK’s National Institute for Health and Clinical Excellence
PANSS: Positive and Negative Syndrome Scale
QTc: QT interval
RCT: Randomized controlled trials
RRs: Risk ratios
SANS: Scale for the Assessment of Negative Symptoms
UEBMI: Urban Employee Basic Medical Insurance
WMDs: Weighted mean differences
